# Tailoring SnO_2_ Defect States and Structure: Reviewing Bottom-Up Approaches to Control Size, Morphology, Electronic and Electrochemical Properties for Application in Batteries

**DOI:** 10.3390/ma16124339

**Published:** 2023-06-12

**Authors:** Reynald Ponte, Erwan Rauwel, Protima Rauwel

**Affiliations:** 1 Institute of Forestry and Engineering, Estonian University of Life Sciences, 51006 Tartu, Estonia; reynald.ponte@emu.ee; 2 Institute of Veterinary Medicine and Animal Sciences, Estonian University of Life Sciences, 51006 Tartu, Estonia; erwan.rauwel@emu.ee

**Keywords:** SnO_2_, nanomaterials, synthesis, polymorphism, band gap, defect states, electrochemical properties

## Abstract

Tin oxide (SnO_2_) is a versatile n-type semiconductor with a wide bandgap of 3.6 eV that varies as a function of its polymorph, i.e., rutile, cubic or orthorhombic. In this review, we survey the crystal and electronic structures, bandgap and defect states of SnO_2_. Subsequently, the significance of the defect states on the optical properties of SnO_2_ is overviewed. Furthermore, we examine the influence of growth methods on the morphology and phase stabilization of SnO_2_ for both thin-film deposition and nanoparticle synthesis. In general, thin-film growth techniques allow the stabilization of high-pressure SnO_2_ phases via substrate-induced strain or doping. On the other hand, sol–gel synthesis allows precipitating rutile-SnO_2_ nanostructures with high specific surfaces. These nanostructures display interesting electrochemical properties that are systematically examined in terms of their applicability to Li-ion battery anodes. Finally, the outlook provides the perspectives of SnO_2_ as a candidate material for Li-ion batteries, while addressing its sustainability.

## 1. Introduction

The transition to carbon-neutral energy production, storage and use is at the forefront of the EU’s decarbonized energy transition. To that end, lithium-ion batteries (LiBs) are currently at the forefront of energy storage devices because of their high energy density, high specific energy, high output voltage, low self-discharge, wide operational temperature range and rechargeability [1,2,3,4,5]. Even though LiBs serve as an energy source for smaller electronic devices, their performance is still insufficient for certain applications, such as electric vehicles (EV), which require good cyclic performances and low self-discharge. In fact, the choice of the anode material in LIBs is essential in determining the storage capacity or energy density of the battery. Currently, transition metal oxides, e.g., Fe_2_O_3_, Co_3_O_4_, NiO and TiO_2_, and composites of graphite with Sn, Sb and Al have raised interest as new-generation electrode materials [6,7,8]. Among them, Sn-based materials (e.g., SnO_2_, SnS_2_, SnSO_4_, Cu_6_Sn_5_ and Ni_3_Sn_4_) possess a higher specific capacity and a lower potential hysteresis than other transition metal oxides [9,10]. Due to their high conductivity and transparency, SnO_2_ materials are used in solar cells, catalytic supports, solid-state sensors and electrode materials for battery applications. SnO_2_ is also an important n-type wide-bandgap (3.6 eV) semiconductor, and its most stable polymorph is the natural and abundant cassiterite ore that crystalizes in the tetragonal rutile structure (P42/mnm). Additionally, compared to the commercialized graphite anode, SnO_2_ anode materials have demonstrated a much higher theoretical specific capacity of 1494 mAhg^−1^ against the 372 mAhg^−1^ of graphite [2,4,6,7,8,9], proving their applicability in commercial anode materials. In addition, the theoretical specific capacity of SnO_2_ is higher for reversible electrochemical half-cell reactions. Even though the alloying and dealloying of Li with metallic Sn are completely reversible, reduction of SnO_2_ into Sn during lithiation is considered as mainly irreversible. Nevertheless, the theoretical specific capacity still remains high at ~780 mAhg^−1^ owing to the formation of various Li_x_Sn compounds after the first cycle [11,12,13,14]. Both the theoretical calculations [15] and cyclic voltammetry studies [16,17] confirm the presence of these intermediate phases resulting from SnO_2_ lithiation. In addition, the ternary phase diagram of Sn-O-Li [18] also indicates that both the Li_2_SnO_3_ and Li_8_SnO_6_ intermediate phases are likely. Lithiation of metallic Sn presents certain disadvantages, such as a large volume expansion (~200–300%) that creates internal stress at the anode and consequently leads to its degradation [9,13,19].

One way to overcome these irreversible reactions that reduce the specific capacitance of the electrode is by using nanomaterials. In fact, nanomaterials are applied to a wide range of fields, e.g., medicine [20], food [21], the environment [22], textiles [23], cosmetics [24], electronics [25] and energy [26]. As the particle size decreases, the surface-to-volume ratio increases, leading to a high specific surface. Recent studies [5,12,27] have shown that shrinking the SnO_2_ nanoparticle size to less than 11 nm [28] enables the reversibility of the lithiation–delithiation processes. In fact, the large surface area in very small nanoparticles generates a large number of reactive sites for Li_2_O nucleation on the surface of SnO_2_ that improves inter-diffusion kinetics. On the other hand, Sn-metal nanoparticles tend to enlarge in order to reduce their surface-to-interface energy, i.e., Gibbs free energy, leading to the decrease in the number of active sites for Li_2_O nucleation and impeding the full particle conversion of Sn to SnO_2_. In fact, Sn coarsening is likely to be the major reason for the decay in energy capacity [5,28,29]. In addition, Li_2_O coarsens simultaneously and acts as an electron insulator because of its poor electronic conductivity and, thus, obstructs electron shuttling between electrodes. Therefore, several strategies have been adopted in order to hinder Sn-nanoparticle coarsening [5]. These methods include combining SnO_2_ with carbonaceous materials such as graphite [5,30], graphene [5,31,32] or carbon nanotubes [5,33]. In turn, these strategies promote reversible reactions owing to a better dispersion of nanoparticles in the matrix. While the carbon matrix usually enhances electrical conductivity, incorporating grain boundaries with a hybrid interface improves Li^+^ insertion that then deters the coarsening of Sn nanoparticles. Another strategy consists of alloying with Co metal that generates intermetallic phases [34,35,36] with high theoretical capacities, i.e., up to 851 mAhg^−1^ for CoSn_3_, 796 mAhg^−1^ for CoSn_2_, 663 mAhg^−1^ for CoSn and 569 mAhg^−1^ for Co_3_Sn_2_ [37].

SnO_2_ can also be grown as hierarchical structures, such as nanorods, nanowires and nanoflowers, with a high surface-to-volume ratio. These morphologies tend to reduce the strain and coarsening of SnO_2_ caused by repeated lithiation–delithiation processes owing to their high-aspect ratio and specific surface that provide ample sites for Li_2_O lithiation. Even though there are reports on their syntheses [1,2,38,39,40,41,42], controlling the final morphology via synthesis parameters still remains a challenge. Furthermore, SnO_2_ bandgap varies as a function of the crystal structure and defects, and both depend upon the synthesis conditions [2,3]. Several high-pressure polymorphs of SnO_2_ possess interesting electronic properties; however, stabilizing them as single-phase free-standing nanostructures has not yet been achieved. Nevertheless, thin-film deposition methods have introduced novel ways to achieve the stabilization of these high-pressure polymorphs owing to substrate-induced strain and dopants [4,5,7,8,43]. For instance, the pure orthorhombic phase is mainly stabilized in SnO_2_ thin films via chemical deposition methods resulting in an epitaxial growth induced by substrate strain. On the other hand, the pure cubic phase was only achieved by direct-current sputtering using nitrogen and antimony dopants [44].

Therefore, this review compiles SnO_2_ synthesis methods and correlates them to the structural, morphological, electronic and optical properties. In fact, the bandgap, crystal structure, morphology and defects can be controlled by the synthesis conditions, such as synthesis temperature, duration, precursor and solvent. These properties are then further correlated to the electrochemical properties, such as energy capacity, redox mechanisms and cyclability of the anode materials, that are compared to commercial batteries. This paper aims at providing a reliable survey of the state of the art on the control of SnO_2_ properties. 

## 2. Stabilization of SnO_2_ Polymorphs

**Figure 1:**
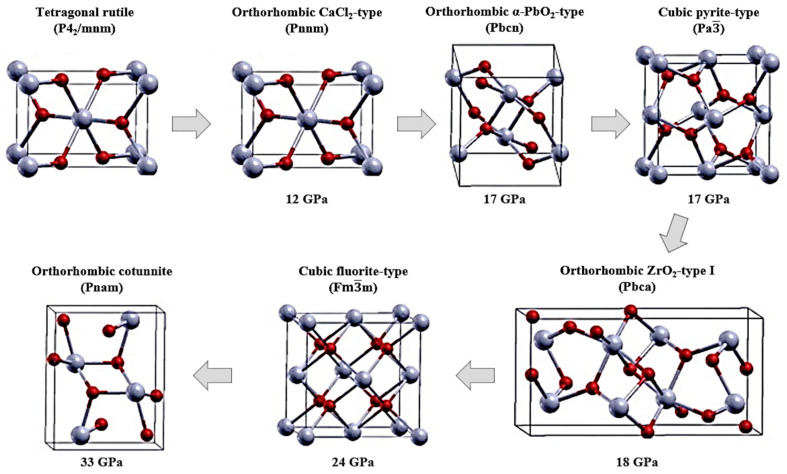
Crystal structures of pressure-induced SnO_2_ polymorphs. Adapted with permission from [51], 2007 American Chemical Society.

The tetragonal rutile structure (P42/mnm) is the most common polymorph of SnO_2_. Other polymorphs of SnO_2_ have been stabilized by various growth techniques and through the use of dopants. Furthermore, the insertion of dopants not only modifies its crystal structure but also its physical and chemical properties. However, to date, the electrochemical performance for each SnO_2_ phase has not been systematically quantified for battery applications. Polymorphs of SnO_2_ vary in their polyhedral stacking and are a result of pressure-induced phase transitions of SnO_2_, shown in Figure 1, starting from the rutile structure, studied by the density functional theory (DFT) [45,46]. In fact, SnO_2_ undergoes a phase transition from the rutile-type tetragonal P42/mnm to the CaCl_2_-type orthorhombic *Pnnm* phase (Figure 1). The *Pnnm* polymorph of SnO_2_ usually stabilizes at a pressure of 12 GPa but can also exist in its metastable form of α-PbO_2_-type orthorhombic or the scrutinyite structure belonging to the space group of *Pbcn* [47]. The scrutinyite phase is under-stoichiometric in oxygen and is produced by an oxygen-vacancy-mediated transformation that not only increases the unit cell volume but also provides them with interesting physical properties, such as enhanced gas sensing. Oxygen vacancies can also be created by introducing trivalent dopants that substitute Sn in the structure, whereupon generating oxygen vacancies in the structure. For example, both Co and Mn are capable of stabilizing the scrutinyite phase, as they possess several oxidation states [43,48]. However, single-phase scrutinyite SnO_2_ is difficult to stabilize and often exists as a mixture of the rutile and orthorhombic *Pnnm* phases. Subsequently, the transformation of scrutinyite into the pyrite-type cubic *Pa*$\bar{3}$ occurs at 17 GPa. Several works claim that high-pressure cubic phases were in fact stabilized from a pressure of 21 GPa onward. Similarly, pressures as high as 48 GPa were used to stabilize the *Pa*$\bar{3}$ phase with a lattice constant of a = 4.87 Å. However, theoretically, a phase transition from *Pa*$\bar{3}$ to fluorite-type cubic *Fm*$\bar{3}$*m* was obtained at a lower pressure of 24 GPa. The differences in the pyrite- and fluorite-type cubic phases lie mainly in the oxygen co-ordination. In the pyrite cubic structure, the Sn atoms are coordinated to six oxygen atoms and two more situated further away. On the other hand, in the fluorite structure, eight oxygen atoms are coordinated equidistantly from Sn. The release of pressure reverses the phase transformation to orthorhombic and then to tetragonal [49]. However, at a lower pressure of ~28 GPa, a ZrO_2_-type I orthorhombic phase transformation at 18 GPa from the pyrite structure belonging to the *Pbca* space group is stabilized, along with a 2% increase in the volume of the unit cell. The volume expansion is due to the presence of a higher number of Sn^+4^-coordinated oxygen, which increases to 7. Similarly, the fluorite-type cubic *Fm*$\bar{3}$*m* has a Sn^+4^ cation coordinated to eight oxygen anions. Finally, the cotunnite-type orthorhombic phase II with a *Pnam* space group appears at 33 GPa with the Sn cation being coordinated to nine oxygen anions [45,49,50].

Although the formation of the orthorhombic phase usually requires high pressure, i.e., extremely energetic conditions, orthorhombic SnO_2_ can nevertheless be stabilized as thin films by controlling the deposition conditions, such as temperature and pressure, or through doping. Several physical deposition techniques are reported in the literature, such as sputtering [52] and pulsed laser deposition (PLD) [53,54], in addition to chemical techniques, such as plasma-enhanced atomic layer deposition (PE-ALD) [55,56] or mist chemical vapor deposition (CVD) [57]. The addition of transition-metal ions or rare-earth-ion dopants has also shown promising results in the stabilization of the orthorhombic phase. In general, comparable-sized or smaller-radius metal ions usually substitute the Sn^4+^ cation in the lattice. For instance, manganese (Mn^3+^ radius: 0.65 Å and Mn^4+^ radius: 0.54 Å) [43], zinc (Zn^2+^ radius: 0.74 Å) [58] or cobalt (Co^2+^ radius: 0.58 Å) [59] ions, depending on their oxidation number, possess very similar atomic radii to Sn^4+^ ions (0.69 Å). Nevertheless, larger-radius ions can also stabilize the orthorhombic phase. In fact, ionic radii of Ce^4+^ (0.87 Å) and Ce^3+^ (1.01 Å) are much larger than the Sn^4+^ ionic radius; however, via the generation of lattice disorders and structural defects, the orthorhombic phase with a molar content of 41% can be stabilized in the solid solution of Sn_0.7_Ce_0.3_O_2_ [60]. Other than lattice disorders, orthorhombic phase stabilization can be obtained by doping SnO_2_ with Zn^2+^, Mn^3+^, Co^2+^ or Ce^3+^, they having lower oxidation states that trigger the formation of oxygen vacancies in the structure and, in turn, trigger the stabilization of other high temperature phases. For smaller cations such as Mn^3+^, the lattice distortions induced by changes in bond length are responsible for stabilization of the lower symmetry orthorhombic phase of SnO_2_ [43]. However, in the case of Sb doping, since Sn and Sb have similar atomic radii, Sb substitutes Sn without modifying the crystal structure even though they have different valences [33]. The presence of Sb^3+^ creates oxygen vacancies in the structure; nevertheless, a critical number of these oxygen vacancies is needed to stabilize the cubic phase.

The SnO_2_ cubic structure, i.e., pyrite or fluorite types, is a higher-pressure phase than the orthorhombic ones. In order to stabilize these phases, the substitution of oxygen by nitrogen atoms in epitaxially grown SnO_2_ films has been carried out [44,61]. The mechanism, once again, is based on oxygen-vacancy creation, where a N^3−^ anion substitutes an O^2−^ anion, creating an oxygen-deficient structure. Meanwhile, the co-doping with Sb^3+^ cations further exacerbates the formation of oxygen vacancies [44].

Even though many studies describe the stabilization of other SnO_2_ metastable phases via thin-film deposition techniques, the synthesis of orthorhombic or cubic SnO_2_ phases remains a challenge. In addition, other metal oxides also show a similar behavior. For example, the stable polymorph of HfO_2_ at room temperature is the monoclinic phase, but the tetragonal and cubic phases can be stabilized by varying certain synthesis parameters, e.g., doping and defects [62]. On the other hand, a reductive atmosphere at a synthesis temperature of 300 °C tends to induce oxygen vacancies, enabling the synthesis of the HfO_2_ cubic phase without dopants [63]. To the best of our knowledge, there are no reports available describing the stabilization of high-pressure single-phase SnO_2_, i.e., cubic or orthorhombic at atmospheric pressure, as these usually exist as mixed phases.

## 3. Electronic Structure of SnO_2_

**Figure 2:**
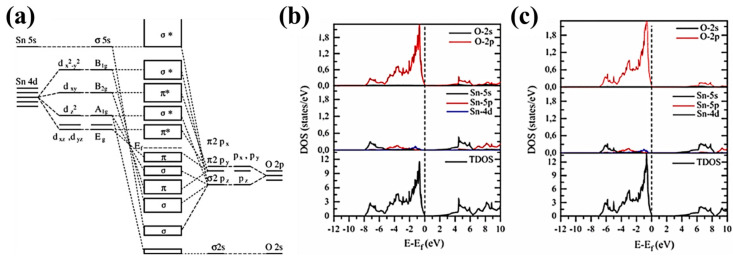
(**a**) Molecular-orbital bonding structure, 2010 Elsevier [70], partial and total density of states calculated by (**b**) generalized gradient approximation by Perdrew et al. [71] (GGAP–PBE) and (**c**) Tran and Blata [72] modified Becke and Johnson [73] potential (Tb–mBJ), 2017 Springer [74] for rutile SnO_2_.

Bulk SnO_2_ has a bandgap of ~3.6 eV; however, experimental bandgaps range from 1.7 eV to 4 eV, thereupon widening its range of applications to photovoltaics and photocatalysis [42,64,65]. Bandgap engineering is widely studied in SnO_2_, as it belongs to the family of transparent conducting oxides (TCO). Additionally, bandgaps can be controlled via parameters, such as synthesis routes and the application of a substrate-induced strain [66] for thin-film growth that simultaneously produce intrinsic defects and structural changes. First-principles calculations indicate that the bandgap can be narrowed by increasing the distortion in the SnO_6_ octahedra, provoking changes in the bond length and bond angles in the unit cell [67]. The molecular-orbital bonding diagram of Figure 2a illustrates the hybridization of B_2g_, A_1g_ and E_g_ with π2p_y_ and σ2p_z_ between Sn-O. The density of states in Figure 2b,c provides the available states that the electrons occupy in the valence and conduction bands. The bottom of the conduction band is mostly the result of the contribution of Sn-4d orbitals with a hybridization of O-2p orbitals. Furthermore, the valence band can be divided into three parts, where the lower part of the valence band corresponds to O-2s and Sn-5s hybridization, the middle part consists of O-O interactions of O-2p, and hybridization of O-2p orbitals with Sn-5p and Sn-4f and the upper part is formed by O-2p and Sn-3d and Sn-4f orbitals [68,69]. Since six atoms of oxygen are at the apices of the octahedra, there are also O-O interactions in the molecular-orbital bonding diagram, in addition to Sn-O interactions. The completely filled Sn-4d and the partially filled O-2p orbitals define the antibonding character of the upper part of the valence band. On the other hand, the middle part has Sn-5p states with fewer than one electron; therefore, all the hybridization interactions with O-2p orbitals possess the bonding character. The presence of O or Sn vacancies affects the bonding character, creating distortion of the unit cell, which in turn modifies the bandgap as explained below. 

Experimental and theoretical results have demonstrated that SnO_2_ possesses both a direct and indirect band gap [42,75,76]. The allowed direct transition corresponds to a 3.68 eV direct bandgap, while the two forbidden direct transitions correspond to 3.03 eV and 3.50 eV [42]. In addition to direct bandgap transitions, there exist also indirect bandgaps corresponding to indirect transition of 2.62 eV and 2.90 eV, respectively [42]. In fact, the fundamental band gap of SnO_2_ is estimated to be much lower at ~3 eV [77], but certain band-to-band optical transitions are dipole forbidden, which lead to a higher optical bandgap of 3.8 eV [78]. In summary, the bandgaps, i.e., direct and indirect, are affected by the presence of defects in the volume of the SnO_2_ structures and the distortion of the oxygen polyhedron owing to polymorphism, as well as surface defects generated as a result of size reduction. This implies that certain transitions that are forbidden in bulk SnO_2_ may be allowed in defective or nano SnO_2_ because of the breaking of the long-range ordering of the crystal lattice at the surface of the nanoparticle [79], which in turn favors the generation of bandgap states.

### 3.1. Bandgap Engineering in SnO_2_

Direct bandgaps are systematically located at the high-symmetry Γ point. Besides, the crystalline quality of thin films related to defects and impurities has been shown to influence the bandgap [80]. The phase transition of SnO_2_ to higher-pressure-induced phases also encourages a decrease in the direct bandgap, which is the result of a more compact lattice, ensuing higher orbital overlapping [81]. In agreement with these observations, Table 1 compiles the structural and electronic properties of all the SnO_2_ polymorphs. However, experimentally, the synthesis and stabilization of higher-symmetry SnO_2_ polymorphs is complicated. Few studies report the presence of other SnO_2_ phases for Fe-doped SnO_2_, owing to the substitution of Sn by Fe ions [82], where the rutile phase co-exists with a small amount of α-PbO_2_ (*Pbcn*) secondary phase. After annealing at 800 °C, they observed that only traces of the SnO_2_ orthorhombic phase remained, which confirms the low stability of the orthorhombic phase at high temperatures compared to the SnO_2_ rutile structure. In addition, the possible presence of iron oxide lowers the SnO_2_ band gap to 2–3 eV [83]. In fact, according to the crystal field theory, Fe^3+^ ions are placed in an octahedral configuration in the presence of a weak field ligand (oxide) in a high-spin configuration. In addition, Fe^3+^ ions possess an ionic radius (0.645 Å) that is slightly smaller than Sn^4+^ ions (0.69 Å), which decreases the lattice parameters and, consequently, increases orbital overlapping between Sn^4+^ and O^2−^ ions, leading to a lower bandgap. Radaf et al. succeeded in stabilizing the orthorhombic SnO_2_ structure by adding Cr^3+^ dopant [84]. The crystallite size decreased with the increase in Cr concentration, and the bandgap consequently decreased from 3.6 eV for the undoped SnO_2_ thin film to 3.28 eV with 5% of Cr. While the addition of those metals leads to the stabilization of the orthorhombic phase, Keskenler et al. [85] have demonstrated that W incorporation also stabilizes the *Pbca* cubic phase until a doping threshold of 2.0 at. %. Here, W^6+^ is likely to substitute Sn^4+^, which shrinks the lattice owing to the lower ionic radius. When the W concentration exceeds 2.0 at. %, lower oxidation states of tungsten could also substitute Sn^4+^ sites, which counteract the unit-cell shrinkage [85]. This can be explained by the Moss–Burstein effect, where materials with high carrier concentration, such as W, fill unoccupied states deep within the conduction band. Consequently, the Fermi level of the n-type SnO_2_ shifts into the conduction band. The increase in the optical bandgap is due to the excited electrons transitioning from the valence band to empty states in the conduction band localized at higher energy levels [85,86]. 

### 3.2. Point-Defect Engineering in SnO_2_

The n-type conductivity of undoped rutile SnO_2_ materials can be explained by the defects present in the structure. Among the four different intrinsic defects, i.e., oxygen vacancy V_O_, tin interstitial Sn_i_, tin antisite Sn_O_ and oxygen interstitial O_i_, the predominant and combined occurrence of V_O_ and Sn_i_ leads to electron donor properties [50,89]. SnO_2_ nanostructures exist in diverse morphologies (e.g., nanorods, nanocubes, nanosheets, nanowire and nanospheres) as a result of the synthesis route. Interstitial and vacancy-mediated defects are specific to each morphology, as the shape and size of the nanoparticle influence the surface and volume defects generated [90,91]. Hence, engineering SnO_2_ nanoparticles via controlled synthesis conditions allows the tailoring of their size, shape, morphology, intrinsic and surface defects. These properties play an important role in their electrochemical properties and redox mechanisms, especially for LiB applications. Defect engineering in semiconductors, more particularly in nanomaterials, is important for several applications. In fact, surface defects in nanomaterials are capital for surface-related phenomena in catalysis. Surface-defect engineering of SnO_2_ has already been studied for photocatalytic [92] and gas sensing [93,94] applications. Furthermore, oxygen-related defects generated in an oxygen-poor environment create exposed Sn^4+^ cations, as well as oxygen vacancies at the surface leading to abundant reactive sites [95]. In general, point defects such as surface-oxygen vacancies are common in nanomaterials owing to the high surface-to-volume ratio [96,97]. These defects are tailored via synthesis conditions, i.e., oxygen-rich or oxygen-poor conditions, synthesis temperature and annealing atmospheres. In addition, synthesizing faceted nanoparticles and exposing certain crystal facets to enhance catalytic activity are important topics in catalysis [98]. Furthermore, doping with foreign atoms to create V_O_ and V_Sn_, as explained before, stabilizes higher-symmetry polymorphs through the production of oxygen vacancies in the structure. On the other hand, in optoelectronic and electronic applications, passivating surface defects is necessary for enhancing the conductivity of SnO_2_, as in the case of F-doped SnO_2_ [99]. The principle is to eliminate defect states within the bandgap of the material by reducing these surface traps and, consequently, increasing the charge mobility and conductivity of SnO_2_. Since an oxygen anion is doubly ionized, the depletion of oxygen leads to a general enhancement of the charge-carrier concentration. These oxygen vacancies, i.e., V_O_, can have three different charge states, commonly termed as neutral V_O_**^x^**, singly ionized V_O_^●^ and doubly ionized oxygen vacancy V_O_^●●^ in Kröger–Vink notation. Two types of stoichiometric defects can occur inside the SnO_2_ lattice, i.e., Frenkel and Schottky defects, which do not influence the conductivity of the material, as the stoichiometry remains the same and charges remain in equilibrium. Schottky defects involve the simultaneous presence of charge-equivalent metal vacancies V_Sn_**^⁄⁄⁄^**^⁄^ (quadruply negatively charged Sn vacancy) and oxygen vacancies V_O_^●●^. The mechanism consists of one Sn^4+^ ion leaving its lattice site (Sn_Sn_**^x^**), along with two oxygen atoms leaving their lattice sites (O_O_**^x^**) simultaneously and diffusing within the crystal in order to create charged vacancies, V_Sn_**^⁄⁄⁄⁄^** and two V_O_^●●^, respectively, whereas Frenkel defects are a type of point defect, where Sn_Sn_**^x^** or O_O_**^x^** leaves its original lattice site and occupies an interstitial site. 

Stoichiometric defect mechanisms do not interfere with electronic properties of SnO_2_ nanoparticles, unlike nonstoichiometric defects. Surface defects in bulk materials have an insignificant influence on their physical and chemical properties because of their low proportion. However, surface defects in nanomaterials can drastically change catalytic and electronic properties, as a result of their high surface-to-volume ratio. Formation of V_Sn_**^⁄⁄⁄⁄^**/V_Sn_**^⁄⁄^**, V_O_^●●^/V_O_^●^/V_O_**^x^**, Sn_i_ and O_i_ depends on the oxygen environment during synthesis [100]. In the case of oxygen-deficient SnO_2_ nanomaterials, oxygen vacancies are formed by the transfer of oxygen atoms from their site (O_O_**^x^**) to the ambient because of an oxygen-poor environment during synthesis. This leads to a metastable state where oxygen vacancies are filled with the remaining two electrons of O^2−^ (V_O_^×^) that then maintain the charge neutrality of the structure. However, this intermediate state is still unstable and leads to the subsequent release of electrons into the surroundings. The electrons released during the formation of the ionized oxygen vacancies are transferred to the Sn-5s state of the conduction band and ionize the Sn cation. These vacancies play a critical role as acceptors, whereupon they form new energy levels deep within the bandgap of the material. However, V_O_, as usual, couples with Sn_i_ in oxygen-deficient conditions, whereupon complex defects are formed, i.e., V_O_^●●^ + Sn_i_**^⁄⁄^**, giving rise to the n-type conductivity of SnO_2_ with electron mobility from the Sn_Sn_**^⁄⁄^** to Sn_Sn_**^⁄⁄⁄⁄^** sites. The mechanism for n-type conductivity involves the hybridization of the Sn-5s and O-2p states near such vacancies, facilitating electron transfer from the valence to the conduction band [101]. The only possibility to obtain p-type conductivity is via the introduction of V_Sn_**^⁄⁄⁄⁄^** + 4 h. In the case of Sn-deficient SnO_2_, V_Sn_ are the predominant defects that are created. An opposite reaction occurs where four electrons are taken from the valence band to form holes in order to generate interstitial site Sn_Sn_**^⁄⁄⁄⁄^**. The production of Sn vacancies can be mediated in Sn-poor conditions or by doping SnO_2_ with tri-valent elements substituting the Sn_Sn_^×^ that create V_Sn_ [102,103]. Simultaneous doping with elements, such as N, creates acceptor states that then facilitate p-type conductivity. The computational and experimental studies on co-doping suggest the replacement of approximately four Sn atoms by four Al atoms and one O atom by one N atom [104]. In the case of metal excess, the defect equation governed by this mechanism involves the formation of Sn interstitial atoms, Sn_i_**^x^**, which further act as electron donors and can be successively doubly ionized to Sn_i_^●●^ or quadruply ionized to Sn_i_^●●●●^. These doubly ionized Sn^2+^ states can act as traps that restrict the possibility of transition from the conduction band minimum to holes just above the valence band maximum. Therefore, passivation of the Sn_i_^●●●●^ on the surface of the SnO_2_ nanoparticles tends to enhance the oxygen-vacancy-related transitions. Lastly, in oxygen-rich conditions, O_i_^⁄⁄^ have the lowest formation energy and are therefore abundant.

**Figure 3:**
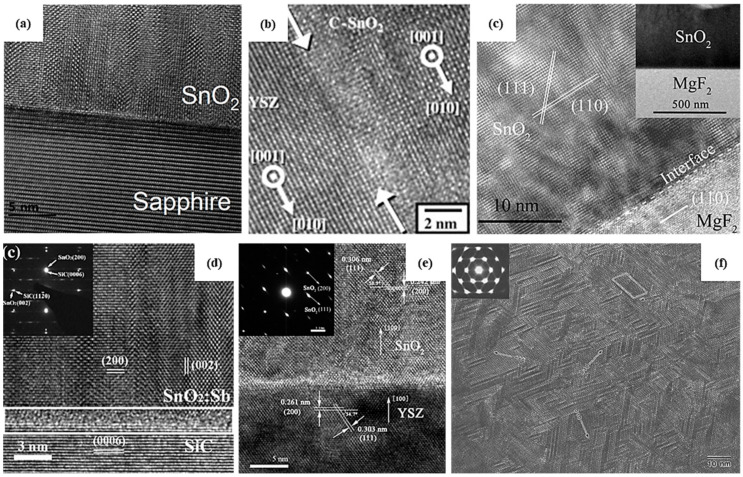
HRTEM images of thin films of orthorhombic SnO_2_ (**a**) on c-plane sapphire by RF sputtering, 2022 Elsevier [117], (**b**) on yttrium-stabilized zirconia (YSZ) substrate by PE-ALD, 2012 Elsevier [55], (**c**) of rutile SnO_2_ on MgF_2_, 2018 Elsevier [123], orthorhombic SnO_2_ on (**d**) 6H-SiC substrate, 2021 Springer Nature [124], and (**e**) YSZ substrate, 2010 Elsevier [125], both by MOCVD; (**f**) Plane-view TEM image of SnO_2_ thin film deposited by MOCVD, 2011 Springer Nature [126].

Among all these point defects described above, oxygen vacancies caused by oxygen-poor conditions are the most abundant intrinsic defects occurring in SnO_2_ nanomaterials because of the lowest formation enthalpy [100]. Moreover, many studies [105,106,107,108,109,110] have probed these new energy levels via photoluminescence (PL) spectroscopy. As previously mentioned, unstable V_O_^×^ vacancy acts as a donor level and is located at 0.03 eV, just under the conduction band. In addition, ionized V_O_^●^ is also considered a shallow donor, as it is located 0.15 eV below the conduction band, while V_O_^●●^ is an acceptor level located at 1.4 eV above the valence band [109,110]. In SnO_2_ nanomaterials, the surface-oxygen vacancy is doubly ionized (or V_O_^●●^) and is the most dominant emission [111]. Wang et al. have investigated the photoluminescence mechanisms under a 255 nm excitation wavelength, resulting in band-to-band and defect excitations. Each peak was successfully identified and energy levels in the band diagram of SnO_2_ also corroborate them. For example, the transition between the V_O_**^x^** donor level to the V_O_^●●^ acceptor level is attributed to the 467 nm (2.65 eV) emission peak, whereas the electron transition from V_O_^●^ to the valence band can be assigned to the 439 nm (2.83 eV) emission peak. In addition, Sn_i_**^x^** is a shallow donor, as Sn interstitials tend to occur exclusively in the +4 state located very near the conduction band, contributing to the n-type semiconductor properties of SnO_2_, even though Sn interstitials are not abundant. The band-to-band transition is identified by the 328 nm emission, corresponding to an energy of 3.78 eV. Habte et al. [112] have demonstrated that the addition of Zn^2+^ cations to the SnO_2_ lattice leads to PL emission peak shifts, shown in Figure 3d,e, toward lower energies. There could be two reasons for the optical bandgap reduction. Since Zn^2+^ cations are smaller, their insertion should promote orbital overlapping because of a reduction in the lattice parameter. The other reason could be the shift toward longer wavelengths corresponding to the ZnO bandgap (3.37 eV). However, they highlighted that the lattice structure remains unchanged with Zn^2+^; therefore, the decrease in the optical bandgap can be attributed to the presence of Zn-O complexes. Salem et al. [113] have observed similar changes in the bandgap with Ni-doped ZnO. Nevertheless, the addition of Zn^2+^ should also enhance emission peak intensities, since the substitution of a smaller and lower valency cation encourages the formation of oxygen vacancies.

Since SnO_2_ is an n-type intrinsic semiconductor, the most prominent defects are, therefore, V_O_ and Sn_i_ because of the lowest formation enthalpy. They are present in the volume of the material and contribute to the electronic conductivity. In nanomaterials, these defects are present on the surface and are instrumental in several catalytic reactions, including oxygen evolution reaction, hydrogen evolution reaction, gas sensing or electrocatalytic CO_2_ reduction [114]. On the other hand, for applications in electronic devices, these surface states are detrimental to the device’s functional properties. Photoluminescence spectroscopy is commonly used to identify these defects by providing information on optical transition between defect levels and band edges [115]. Depending on the application, these surface defects need to be either passivated or exacerbated. The importance of doping SnO_2_ with acceptors lies in the possibility of obtaining a p-type semiconductor that would eventually lead to a SnO_2_ homojunction diode. In general, surface defects act as trap states that enhance defect-level emission from the bandgap states. Furthermore, these defect states also extend the photo absorption of the materials to the visible region. Consequently, several new applications in LED, visible light detectors and photocatalysis are likely. In addition, defect-induced structures lead to the stabilization of higher-pressure polymorphs of SnO_2_, further leading to bandgap variations and other interesting properties, as explained in the section below.

## 4. Synthesis of SnO_2_ Nanostructures: Thin Films, Nanoparticles and Nanocomposites

### 4.1. Thin-Film Growth of SnO_2_: Role of Substrate-Induced Strain in the Stabilization of High-Pressure Phases of SnO_2_

Several studies report the stabilization of different phases of SnO_2_ as thin films through the optimization of growth conditions. In fact, epitaxial growth can promote the stabilization of high-pressure phases of SnO_2_ through substrate-induced strain [66]. Table 2 resumes SnO_2_ thin-film growth parameters by physical and chemical vapor deposition techniques along with the phase stabilized. Physical vapor deposition (PVD) techniques demonstrate some advantages over chemical deposition techniques as different polymorphs of SnO_2_ as thin films can be grown through PVD more easily. For PLD, the most important parameter is the deposition temperature [53,54,116]. At low temperatures (~150–300 °C), thin films are mainly amorphous because of low atomic mobility [54,116]. At a higher temperature (700 °C), orthorhombic and tetragonal phases coexist; however, at very high temperatures (1150 °C), the atomic diffusion is extremely high and SnO_2_ therefore tends to rearrange itself in its most stable structure, i.e., rutile [54]. Similarly, for DC and RF sputtering, authors demonstrated that it is possible to stabilize the rutile, orthorhombic or cubic phases depending on the synthesis parameters. Ham et al. [117] succeeded in growing a polycrystalline thin film with a co-existence of the orthorhombic and tetragonal phases. A small amount of rutile SnO_2_ at the interface diminishes the mismatch strain to 0.42%, and in turn, the remaining substrate-induced strain enables a heteroepitaxial growth of orthorhombic SnO_2_ on the c-plane of sapphire. The high-resolution TEM image in Figure 3a reveals a smooth interface between the substrate and the film oriented [11,12,13,14,15,16,17,18,19,20] and [001], respectively, implying that the growth direction of tetragonal-phase SnO_2_ is <100>. Whereas the orthorhombic phase can be obtained by sputtering at a high temperature [117,118], the cubic phase is mainly obtained by substituting O atoms by N atoms with N_2_ gas [44,61,119]. Similar to other metal oxides, such as CeO_2_ [120], ZrO_2_ [121] and HfO_2_ [122], SnO_2_ can be doped with nitrogen, where the substitution of O by N atoms involves an increase in ordered oxygen vacancies within the crystal structure. By virtue of the epitaxial strain at the interface of SnO_2_ and the substrate, crystallization of the orthorhombic or cubic phase can occur. However, at exceedingly high temperatures, the stabilization of the orthorhombic or cubic phase is hindered and the tetragonal rutile phase, where the Gibbs free energy is the lowest, is promoted [54]. 

Only a few reports of chemical deposition techniques describing the stabilization of the orthorhombic SnO_2_ phase are available. Bae et al. [57] have successfully grown the tetragonal and orthorhombic structures via mist-CVD, using two different solvents, i.e., methanol and acetone. The difference in the boiling point of these two solvents favors the stabilization of one phase over the other. As acetone has a lower boiling point, it would supply oxygen atoms more readily than methanol, leading to oxygen-rich conditions. Their results from DFT calculations were also consistent with the experimental results [57], and they concluded that the SnO_2_ orthorhombic phase is thermodynamically more favorable under Sn- and O-rich conditions. Another deposition using plasma-enhanced atomic-layer deposition for epitaxial growth of the orthorhombic phase by Kim et al. [55] on an yttrium-stabilized zirconia (YSZ) substrate (Figure 3b) with a lattice mismatch of 2% or less has been realized. Furthermore, the deposition of SnO_2_ thin films using metal–organic chemical vapor deposition techniques has also been investigated on different substrates. Deposition on both sapphire and MgF_2_ (Figure 3c) substrates leads to the tetragonal rutile SnO_2_ structure [55,123,127]. On the other hand, the orthorhombic phase is the result of the deposition at similar temperatures (around 500 °C) but on different substrates, i.e., 6H-SiC (Figure 3d) or yttrium-stabilized zirconia (YSZ) (Figure 3e) [124,125]. Furthermore, in that work, Sb doping was responsible for the stabilization of the orthorhombic structure. However, phase stabilization is unlikely because of Sb doping alone, which creates oxygen vacancies, but is also the result of substrate orientation, enabling the epitaxial growth of SnO_2_, owing to the equivalent crystallographic properties of the substrates. Liu et al. have demonstrated that the grain size of epitaxially grown SnO_2_ via MOCVD (Figure 3f) can be controlled by varying the lattice mismatch between the substrate and the film through substrate orientation. Furthermore, Kong et al. report that the [100] interplanar spacing of orthorhombic SnO_2_ film and YSZ substrate are comparable, with a lattice mismatch between c and a lattice parameter of SnO_2_ and YSZ being equal to 1.3% [125], which allows epitaxial thin-film growth.

**Table 2:** SnO_2_ thin-films growth by physical and chemical deposition techniques and their structural properties.

Deposition Technique	Target/Precursor	Oxygen Supply	Substrate	Conditions	Structure	Ref.
Physical deposition methods						
Pulsed laser deposition (PLD)	Rutile SnO_2_	O_2_	Si [100]	308 nm 10 Hz 20–400 °C <10^−5^ Pa	Amorphous + Tetragonal rutile	[116]
PLD	Sintered rutile SnO_2_	Target	Si [001]	532 nm 5 pulses/s 20–1150 °C 4 h	Tetragonal rutile + Orthorhombic	[54]
PLD	Rutile SnO_2_	Target	Si [100]	248 nm 10 Hz 320 °C 3 × 10^−2^ Pa	Tetragonal rutile + Orthorhombic	[53]
Direct-current (DC) sputtering	Tin metal plate	O_2_	Si [100]	Ar gas 40–60 W 5 × 10^−5^ Pa 550 °C	Tetragonal rutile	[128]
DC sputtering	Rutile SnO_2_	Target	SiO_2_	N_2_-Ar gas 15 W 5 × 10^−1^ Pa 20–500 °C	Tetragonal rutile + cubic	[119]
DC sputtering	Tin metallic disk	O_2_	Si [100]	Ar gas 60 W 40 min 1 × 10^−1^ Pa 148–243 °C	Tetragonal rutile + Orthorhombic	[118]
DC sputtering	Sb_2_O_3_ doped rutile SnO_2_	Target	Si [100]	N_2_-Ag gas 60 W 4 × 10^−1^ Pa 300 °C	Cubic	[44]
Radio-frequency (RF) sputtering	Pure metallic Sn	O_2_	SiO_2_	Ar gas 25 W 2 h	Tetragonal rutile	[129]
RF sputtering	Rutile SnO_2_	Target	Sapphire (0001)	50 W 6.67 × 10^−4^ Pa 600 °C	Tetragonal rutile + Orthorhombic	[117]
RF sputtering	Rutile SnO_2_	Target	Si/SiO_2_ + MgO [001]	N_2_-NH_3_ gas 25 to 75 W 0.67 Pa 400 °C	Cubic	[61]
Chemical deposition methods						
Plasma-enhanced atomic-layer deposition (PEALD)	SnCl_4_	O_2_	Si [100]	Ar gas 100 to 400 W 400 Pa 150–350 °C	Tetragonal rutile	[130]
PEALD	Dibutyl tin acetate	O_2_	yttria-stabilized zirconia	Ar gas 100 W 2.67 Pa 300 °C	Orthorhombic	[55]
PEALD	dimethylamino-2-methyl-2-propoxy-tin(II)	O_2_ or H_2_O	Si	N_2_ gas 100–300 °C	Tetragonal rutile + orthorhombic	[56]
Combustion vapor deposition	tin(II) 2-ethylhexanoate in absolute ethanol	Precursor	SiO_2_	20 min 850 °C	Tetragonal rutile	[131]
Aerosol-assisted chemical vapor deposition	Mg_x_Sn_1−x_O_2_ in ethanol	Precursor	Glass	Ar gas 30 min 400 °C	Tetragonal rutile	[132]
Metal–organic chemical vapor deposition (MOCVD)	Dibutyl tin acetate	O_2_	Sapphire (0001)	N_2_ gas 2667 Pa 600–700 °C 30–100 sccm	Tetragonal rutile	[126]
MOCVD	Tetraethyl tin	O_2_	MgF_2_ (001)	N_2_ gas 50 sccm 2 h 540–660 °C	Tetragonal rutile	[127]
MOCVD	Tetraethyl tin and trimethylstibine	O_2_	6H-SiC (0001)	N_2_ gas 40 sccm 2 h 600 °C	Orthorhombic	[124]
MOCVD	Dibutyl tin acetate	O_2_	yttria-stabilized zirconia (100)	N_2_ gas 30 sccm 500–600 °C	Orthorhombic	[125]
Mist chemical vapor deposition	SnCl_2_·2H_2_O in methanol	Precursor	Si	1.5 MHz N_2_ gas 1000 sccm 250–300 °C	Tetragonal rutile	[57]
MCVD	SnCl_2_·2H_2_O in acetone	Precursor	Si	1.5 MHz N_2_ gas 1000 sccm 350–400 °C	Orthorhombic	[57]

### 4.2. Hydrothermal Synthesis of SnO_2_ Nanomaterials

**Figure 4:**
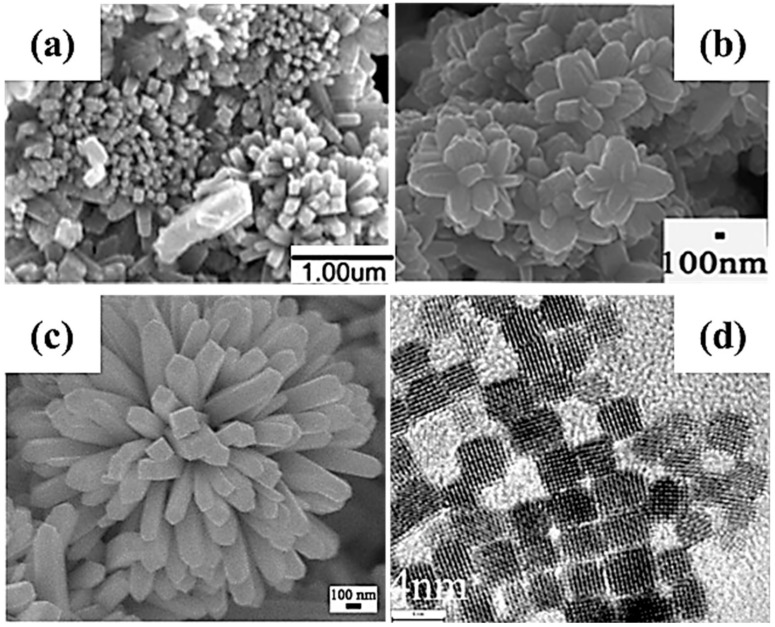
FESEM image of the SnO_2_ nanostructure synthesized with a (**a**) SnCl_4_·5H_2_O:NaOH molar ratio of 1:7.8, 2011 Elsevier [134], (**b**) SnCl_4_:NaOH molar ratio of 1:8 (with PEG), AIP publishing [135] and (**c**) 10:1 molar ratio of SnCl_4_:NaOH, 2010 Elsevier [137]. (**d**) HRTEM image of SnO_2_ nanocubes synthesized with SnCl_4_, urea and fuming urea with a synthesis time of 15 h, 2010 American Chemical Society [138].

The structure–property relationship in SnO_2_ nanomaterials is controlled by the synthesis conditions. This signifies that the bandgap, size of the nanoparticle, morphology and chemical properties can be tuned through the synthesis conditions. Gavaskar et al. [133] have synthesized nanoparticles of SnO_2_ with the rutile structure using a SnCl_4_·5H_2_O precursor in ethanol at 200 °C for 20 h. They obtained quasi-spherical nanoparticles with diameters ranging from 50 to 90 nm and with a direct bandgap (3.35 eV) narrower than most SnO_2_ bandgaps. NaOH is also often used as an oxygen or hydroxyl source, since SnO_2_ crystals grow from stannate Sn(OH)_6_^2−^ that then condense to SnO_2_ and water [134,135,136]. Vuong et al. [134] added an aqueous solution of NaOH to SnCl_4_·5H_2_O dissolved in ethanol, which led to the growth of nanoflowerlike structures in Figure 4a under equivalent synthesis conditions. In addition, SnO_2_ anisotropic crystal growth depends on the diffusion of Sn(OH)_6_^2−^ at the surface of preferred orientations of the rutile crystal structure, which occurs after a sufficient amount of time (24 h) and temperature (190 °C). Guan et al. [135] realized complementary studies, in which they investigated the influence of the ratio of the SnCl_4_·5H_2_O precursor to NaOH on the morphology and structure. At a high ratio of NaOH to SnCl_4_, the nanoparticles are larger compared to low NaOH concentrations. They also explained that a high NaOH concentration promotes the growth of SnO_2_ along preferential crystallographic directions, leading to the flowerlike SnO_2_ nanorod bundles in Figure 4b [135]. Despite having a completely different shape and crystallite size, direct bandgap energies still range from 3.68 eV to 3.72 eV at both low and high NaOH:SnCl_4_ ratios, respectively. The addition of a surfactant, such as PEG [137], has been shown to further improve the previous synthesis protocol by precipitating the uniform flowerlike SnO_2_ nanorod bundles in Figure 4c. 

Cao et al. confirmed the production of nanoflowers under a high NaOH:SnCl_4_ ratio and reduced the synthesis time from 24 h to 12 h [139]. By using sodium dodecyl sulfate catalyst and Na_2_SnO_3_ precursor, they also succeeded in synthesizing SnO_2_ nanocubes by shortening the reaction time to only 4 h, whereupon limiting the growth to preferential crystallographic directions. Runa et al. [140] also managed to synthesize SnO_2_ nanocubes using SnCl_4_ precursor with urea as a surfactant and absolute ethanol as a solvent in an acidic solution. Similar temperatures and time were employed, but an acidic medium and short synthesis time led to a cube morphology. These conditions limit the growth along the thermodynamically preferential [001] direction of SnO_2_ nanorods and lead to a pseudo-cubic shape. Similarly, Xi et al. [138] also synthesized SnO_2_ nanocubes, as shown in Figure 4d, from a SnCl_4_ precursor with urea and HCl. They suggest that hydroxyl ions from the ammonia–water reaction are in equilibrium with ammonium and hydroxide, thus favoring uniform growth morphologies. 

## 5. Prospects of SnO_2_ Nanomaterials as Anode Materials in LiB: Correlating Their Morphology Obtained from Synthesis Routes to Their Electrochemical Performance

Nanocomposites of SnO_2_ have gained attention as anode materials for LiB applications because of their high theoretical specific capacity. It represents one of the most promising anode materials because of its high energy capacity, low cost and high energy density. Among common metal oxide compounds used as anodes in LiB [141], SnO_2_ exhibits the highest theoretical energy capacity (1494 mAhg^−1^), against 890 mAhg^−1^ for Co_3_O_4_ [142], 1007 mAhg^−1^ for Fe_2_O_3_ [143] and 1230 mAhg^−1^ for MnO_2_ [144]. In addition to higher energy capacity and density, SnO_2_ possesses a low overall potential, i.e., charge and discharge voltages of 0.3 V and 0.5 V vs. Li/Li+, respectively [145], against 1.0 and 2.2 for Co_3_O_4_ [146] and 1.3 and 1.6 for MnO_2_ [147]. SnO_2_ nanomaterials could solve issues faced by other metal oxides related to lithium alloying, which leads to irreversible capacity reduction and volume changes [17]. A commercial battery is assessed by different criteria, such as energy density (amount of energy that can be stored per unit mass of the battery), battery power (rate at which the electrical current can be moved through the battery), cycle life (number of charge–discharge cycles until its performance drops), watt hours (amount of power deliverable in an hour), charge speed and resistance or impedance. To solve challenges concerning energy storage systems and their sustainability, measurable quantities are crucial. While energy capacity is a direct indicator of the energy storage performance of the anode, the coulombic efficiency quantifies the reversibility and the efficiency of electron transition from the anode to the cathode over a cycle. Capacity retention evaluates the cycle-life performances of a battery, as it provides the ratio between the discharge energy capacity of successive cycles to the initial one [148]. 

**Figure 5:**
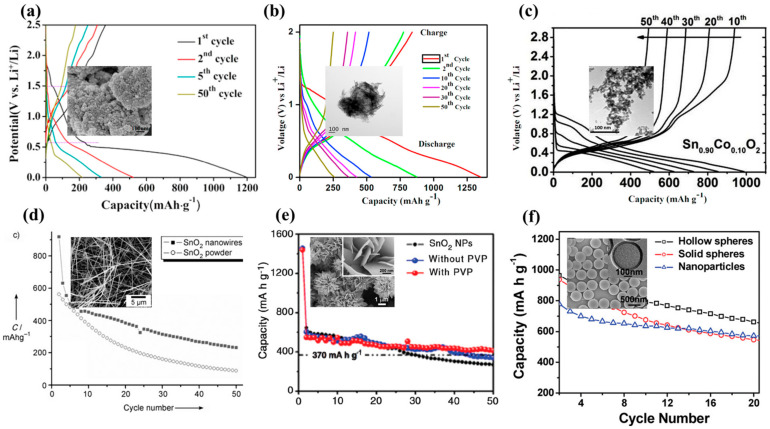
Charge–discharge profiles at 100 mAhg^−1^ current density of (**a**) SnO_2_ nanoparticles, 2016 Elsevier [139], (**b**) SnO_2_ nanosheets, 2018 Elsevier [145] and (**c**) Co-doped SnO_2_ nanoparticles 2018 Elsevier. Cycling performance at 100 mAhg^−1^ current density of (**d**) 1D SnO_2_ nanowire, 2007 Wiley [163], (**e**) 2D SnO_2_ nanosheet, 2012 Elsevier [164] and (**f**) 3D SnO_2_ hollow nanospheres, 2010 American Chemical Society [161]. Insets correspond to their respective SEM or TEM images.

**Figure 6:**
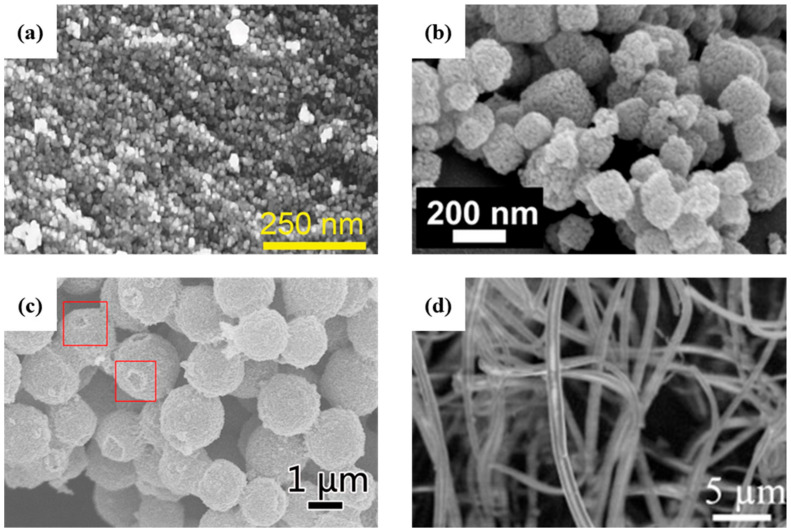
SEM images of SnO_2_ (**a**) porous ordered interconnected, 2014 Wiley [140], (**b**) porous submicrotubes, 2016 Wiley [141], (**c**) porous microspheres, 2018 Elsevier [142] and (**d**) nanotubes, 2010 Elsevier [143].

Hu et al. demonstrated that a spherical nanoparticle with a size under 11 nm is required for a completely reversible lithiation–delithiation reaction [28]. Table 3 summarizes different synthesis routes of SnO_2_ nanostructures and nanocomposites with their electrochemical performances. Hydrothermal methods are a common way to synthesize SnO_2_ nanoparticles because of their low cost and simple synthesis protocols that can be easily scaled up. Yin et al. [149] developed a hydrothermal method based on the utilization of HCl, SnCl_4_∙5H_2_O and ammonia in an aqueous solution heated at 160 °C for 30 min that produced SnO_2_ nanoparticles with sizes ranging from 9 to 21 nm. These nanoparticles display an irreversible discharge capacity of 22.8% after 50 cycles (from 1196.6 mAhg^−1^ to 217.0 mAhg^−1^) with a current density of 100 mAg^−1^ (Figure 5a,d). Although the short synthesis time enables the growth of small nanoparticles, aqueous synthesis does not allow proper control of growth kinetics. On the other hand, nonaqueous synthesis methods are usually preferred for the production of small-sized metal–oxide nanoparticles, as they offer better control of the synthesis and tailor particle shape and size. Etacheri et al. [150] also prepared ultrathin SnO_2_ nanoparticles by reflux in an aqueous solution. High-capacity retention is obtained after calcination of the SnO_2_ nanopowder post-synthesis, referred to as “ordered interconnected SnO_2_ nanoparticles” in Figure 6a vs. pristine materials called “disordered SnO_2_ nanoparticles”. Whereas heat-treated SnO_2_ nanoparticles demonstrate a high-capacity retention (81.9%) and specific capacity (~500 mAhg^−1^) after 100 cycles, pristine SnO_2_ nanoparticles exhibit poor electrochemical properties, i.e., 35.2% of capacity retention and a specific capacity of about 92 mAhg^−1^. Both SnO_2_ nanostructures are agglomerated, but the reason behind the enhancement of their electrochemical properties dwells in the nature of the agglomerates. In fact, aqueous synthesis tends to produce uncontrolled agglomerates with numerous and large electro-inactive clusters, while calcination rearranges the structure into a porous network because of the release of hydroxyl groups. Porous networks are commonly tested for battery applications, such as sub-microtube [151], hollow microspheres [152] and porous nanotube [153] structures (Figure 6b–d), because of their potentially high electrochemical properties. Therefore, reaction reversibility is possible by reducing the lithium-ion diffusion pathway by creating a highly porous structure made of nanorods, nanoflakes, nanobelts and nanosheets [154]. Narsimulu et al. [155] prepared ~10 nm thick SnO_2_ nanosheets by a microwave-assisted synthesis method using a stannic chloride precursor and citric acid in an aqueous medium. In theory, the thin SnO_2_ nanosheets, along with their porous nature, should enable high electrochemical reversibility; however, at a current density of 100 mAg^−1^, the discharge energy density decreases from 1350 mAhg^−1^ to 257.8 mAhg^−1^ after 50 cycles (Figure 6b,e), representing a capacity retention of only 19.1%. In fact, the ordered interconnected SnO_2_ network mentioned before by Etacheri et al. [150] possesses a four-times larger active surface area (204 m^2^g^−1^) than the nanosheets (59.28 m^2^g^−1^). Nano–Köhler theory [156] refers to the activation of inorganic cluster growth by spontaneous condensation [157], but in the case of a prolonged hydrothermal synthesis, it tends to promote a longer coagulation time and, thus, leads to larger nanostructures, such as nanoflower bundles [158] or nanorod arrays [159]. Wen et al. [158] have synthesized flowerlike structures similar to Guan et al. [135] and Cao et al. [139] using NaOH and SnCl_4_∙5H_2_O precursors in aqueous media. However, since cycling performances are carried out at a 10-times lower current density, i.e., 78 mAg^−1^ instead of 782 mAg^−1^, the initial and final energy capacities are not directly comparable. Liu et al. [159] have grown organized nanorods on an Fe plate with similar conditions, using stannic chloride and NaOH precursors heated at 200 °C (vs. 180 °C) for 24 h in an autoclave. In this case, long nanorods with dimensions of 60 nm × 670 nm were produced that exhibited greater initial energy charge and discharge capacities of 1128 mAhg^−1^ and 1918 mAhg^−1^, respectively. On the other hand, for the same current density, nanoflower bundles [158] display charge and discharge energy capacity of 815 mAhg^−1^ and 1673 mAhg^−1^, respectively. Higher electrochemical performances are again due to the nanoarrays presenting a larger organized network facilitating Li^+^ ion diffusion.

Therefore, reaction reversibility is possible on reducing the lithium-ion diffusion pathway, such as in highly porous structures made of nanorods, nanoflakes, nanobelts, nanosheets and hallow nanospheres (Figure 5d–f) [144]. While the synthesis of 0D nanostructures generally requires a SnCl_4_ precursor, hierarchical nanostructures are synthesized by using lower oxidation states, Sn(II), of the SnCl_2_ precursor. Sharma et al. [160] and Ding et al. [161] have, respectively, synthesized 1D SnO_2_ nanowires (Figure 5d) and 3D hollow nanospheres (Figure 5f) using the template-assisted synthesis route. These nanostructures tend to retain their energy capacity for a higher number of cycles compared to free-standing SnO_2_ nanoparticles. Wu et al. [162] and Narsimulu et al. [155] have both, respectively, prepared ~35 nm (Figure 6e) and ~10 nm thick SnO_2_ nanosheets by a hydrothermal method. Both types of nanosheets exhibited good cycling capacity and charge retention.

The coupling of carbonaceous materials with SnO_2_ nanoparticles has been widely studied because of the possible enhancement of cycling capacity and conductivity through their combination. Liu et al. [165] recently illustrated the efficiency of carbon nanotubes and carbon nanotube hairballs coupled with SnO_2_ nanoparticles. These carbonaceous materials and a tin precursor were mixed and stirred in an absolute ethanol (nonaqueous) medium before heating at 150 °C for 10 h. Once the product was washed and dried, the resulting powder was calcinated at 360 °C for 10 min. The initial specific discharge capacity tripled (from 768.1 mAhg^−1^ to 2255.2 mAhg^−1^) when coupled with carbon nanotube hairballs. In addition to higher initial charge and discharge capacities, capacity retention also increases after 100 cycles at 200 mAhg^−1^ from 29.9% (244.8 mAhg^−1^) to 74.2% (809.2 mAhg^−1^), highlighting the high contribution of carbon nanotubes to anode performance. Deng et al. [166] have compiled a large number of syntheses involving SnO_2_ nanostructures coupled with graphene and have evaluated their respective electrochemical performances. The study concludes that SnO_2_–graphene nanocomposites are promising as they could significantly improve electrochemical and electrical properties, even though some issues persist, such as the high cost of graphene fabrication and the large irreversible capacity loss of the initial cycle.

Another solution to improve SnO_2_ properties is through doping with transition metals to enhance the chemical, defect and structural properties of SnO_2_. Mueller et al. [167] measured the electrochemical properties of Fe-doped SnO_2_ nanoparticles in a carbon matrix. Fe-doped SnO_2_ nanoparticles were synthesized via hydrothermal synthesis from tin acetate, sucrose, acetic acid and iron (II) gluconate at 150 °C for 10 h. The doping of SnO_2_ nanoparticles with smaller Fe cations decreases the cassiterite lattice structure through the incorporation of oxygen vacancies, which results in the production of smaller nanoparticles (7 to 8 nm vs. 15 nm). Mueller et al. [167] also illustrate that the Fe-ion doping creates smaller nanoparticles with surface defects. The utilization of a carbon matrix coupled with Fe doping doubles the energy capacity, i.e., from 764 mAhg^−1^ to 1519 mAhg^−1^ after 10 cycles at a current density of 50 mAhg^−1^. This is likely due to the enhanced conductivity of the carbon matrix allowing a better ionic diffusion. In addition, Ma et al. [168] have distinctly quantified the contribution of Co doping on SnO_2_ nanoparticles and the carbon matrix to the system, separately. This synthesis differs from the usual hydrothermal synthesis carried out inside an autoclave. They applied a one-pot synthesis route at 180 °C until complete evaporation occurred, followed by heat treatment at 450 °C for 3 h, which lead to the production of ultra-small nanoparticles doped with Co less than 10 nm in diameter. After 50 cycles at 100 mAg^−1^, Co-doped SnO_2_ nanoparticles show a reversible capacity of 493 mAhg^−1^ (Figure 5c,f), which is higher than undoped SnO_2_ nanoparticles having a reversible capacity of 242 mAhg^−1^. The reversible capacity was then significantly enhanced with the addition of the carbon matrix, where the specific energy capacity remains above 1000 mAhg^−1^. Ou et al. [169] investigated the properties of Ni-doped SnO_2_ nanoparticles synthesized using urea as a surfactant. The protocol requires a SiO_2_ nanosphere template, on the surface of which a thin layer of SnO_2_ nanoparticles is deposited using urea as a surfactant, hindering the excess growth of SnO_2_ despite the long synthesis time of 36 h at 170 °C. The SiO_2_ template is then removed via HCl etching before being calcinated at 400 °C for 4 h under an Ar atmosphere. Although the initial capacity loss is still prevalent after 300 cycles, they showed that Ni doping enhances the specific discharge capacity by more than 600%. SnO_2_ metal–oxide nanocomposites demonstrate significant improvement in the overall electrochemical properties. W. Zhou et al. [170] synthesized SnO_2_-Fe_2_O_3_ nanowire composites that possess a two-times higher energy density than SnO_2_ nanowires. The high-aspect ratio and the compatibility in the electronic structure are responsible for the enhancement. Wang et al. successfully synthesized SnO_2_-graphene-oxide-Co_3_O_4_ nanocomposites that exhibit long cycling stability (641 mAhg^−1^ at 1000 mAg^−1^ after 900 cycles) with complete reversibility (CR = 100%, 1038 mAhg^−1^) after 100 cycles at a lower energy density (100 mAg^−1^) [171]. Both SnO_2_-graphene and SnO_2_-graphene-metal–oxide nanocomposites tend to significantly improve the electrochemical and electrical properties of SnO_2_.

The synthesis of SnO_2_-based nanoparticles is mainly carried out by hydrothermal synthesis routes because of the ability of operating at low synthesis temperatures, including room temperature. Nevertheless, in order to eliminate reaction by-products and improve the properties of the as-synthesized SnO_2_, post-synthesis annealing is usually required in the range of 350–500 °C. Annealing can also induce the formation of porous structures and networks with higher specific surfaces. Other methods such as template-assisted syntheses are also applied to create SnO_2_ porous network nanostructures. In fact, porous connected networks such as SnO_2_ nanotubes provide pathways for Li diffusion, which increases the overall charge retention. In addition, the coupling of SnO_2_ nanostructures with carbonaceous materials, especially graphene, which is a well-known strategy to enhance the electrochemical properties of SnO_2_ nanomaterials, is still limited by the fabrication cost of graphene. For other hierarchical structures, such as nanorods, nanoflowers and nanosheets, an overall enhancement in the electrochemical properties is also observed. Additionally, using dopants in these syntheses, such as Fe, Co and Ni, clearly enhance charge retention and coulombic efficiency. Furthermore, when doped SnO_2_ is combined with carbon-based nanomaterials, its charge capacity is at least doubled. All these strategies starting from simple hydrothermal SnO_2_ synthesis followed by doping and combining with nanocarbons are important technological steps toward increasing the electrochemical properties of SnO_2_. Morphologies exhibiting high surface areas, such as nanoflowers, 3D porous nanostructures or any three-dimensional nanomaterials can promote lithium diffusion within the network owing to the significantly increased surface area. However, 1D and 2D nanomaterials are generally preferred because of their isotropic configuration that enables the stacking of nanomaterials while delivering free active sites.

**Table 3:** Synthesis of SnO_2_ nanocomposites and their electrochemical properties.

Label	Chemicals	Sample Preparation	Shape and Size	Potential Window vs. Li/Li^+^ (V)	Initial Energy Density (mAhg^−1^)	CE/CR	Energy Capacity (mAhg^−1^)	Ref.
SnO_2_	SnCl_4_·5H_2_O, NH_3_, HCl in water	Autoclave at 160 °C for 30 min	Nanospheres 6–21	0.01–2.0	Discharge: 1196.6 Charge: 520 (100 mAg^−1^)	CE = 42% first cycle CE > 98% after 10 cycles CR = 22.8% 50th cycle	217.0 mAhg^−1^ at 100 mAg^−1^ after 50 cycles	[149]
SnO_2_	SnCl_4_·5H_2_O, citric acid in water	Microwave at 2.4 GHz under 160 °C for 30 min	Nanosheets <10 nm thick	0.005–2.0	Discharge: 1350 Charge: 840 (100 mAg^−1^)	CE = 62% first cycle and >97% from the 10th cycle CR = 19.1% 50th cycle	257.8 mAhg^−1^ at 100 mAg^−1^ after 50 cycles	[155]
SnO_2_	Tin(IV) isopropoxide in water	Hydrothermal in air at reflux for 30 min then calcination at 400 °C for 1 h	Nanospheres 9 nm agglomerated in um blocks	0.01–1.5	Discharge: 674.5 (782 mAg^−1^)	CR = 81.9% 100th (782 mAg^−1^)	500 mAhg^−1^ at 782 mAg^−1^ 100th cycle	[150]
SnO_2_	SnCl_4_·5H_2_O, NaOH, oleic acid in water	Hydrothermal, 180 °C for 24 h then annealed for 24 h at 500 °C	Flowerlike nanorod bundles 30 nm	0–2.5	Discharge: 1673 Charge: 815 (78 mAg^−1^)	CE = 49% 1st cycle CR = 41.5% 40th cycle	694 mAhg^−1^ at 78.2 mAg^−1^ after 40 cycles	[158]
SnO_2_	SnCl_4_·5H_2_O, NaOH in water	Hydrothermal, 200 °C for 24 h	Nanorods of 60 by 670 nm	0.005–2.5	Discharge: 1918 Charge: 1128 (78.1 mAg^−1^)	CE = 59% 1st cycle CR = 57.5% after 100th cycle	645 mAhg^−1^ at 78.2 mAg^−1^ after 100 cycles	[159]
SnO_2_	SnCl_2_·2H_2_O in ethanol	Autoclave 150 °C, 10 h then heat treated at 360 °C for 10 min	Micrometric aggregates	0.01–3.0	Discharge: 768.1 Charge: 414.8 (100 mAg^−1^)	CE = 54.0% 1st cycle CE = 93.2% 3rd cycle CR = 29.9% 100th cycle at 200 mAg^−1^	244.8 mAhg^−1^ at 200 mAg^−1^ after 100 cycles	[165]
SnO_2_-CNTH	SnCl_2_·2H_2_O, CNTH in ethanol	Autoclave 150 °C 10 h then heat treated at 360 °C for 10 min	Micrometric hairball shape containing SnO_2_ nanospheres (5–10 nm) and CNTH (30 nm diameter)	0.01–3.0	Discharge: 2255.2 Charge: 1098.3 (100 mAg^−1^)	CE = 48.7% 1st cycle CE = 88.8% 3rd cycle CR = 74.2% 100th cycle at 200 mAg^−1^	809.2 mAhg^−1^ at 200 mAg^−1^ after 100 cycles	[165]
SnO_2_	Sucrose, acetic acid, tin acetate in water	180 °C to evaporate then 300 °C to completely dry then calcinated at 450 °C for 3 h	Nanospheres of 15 nm	0.01–3.0	Discharge: 1139 Charge: 679 (50 mAg^−1^)	CE = 40.4% 1st cycle	764 mAhg^−1^ at 50 mAg^−1^ after 10 cycles	[167]
Fe-doped SnO_2_	Iron(II) gluconate·2H_2_O sucrose, acetic acid, tin acetate in water	180 °C to evaporate then 300 °C to completely dry then calcinated at 450 °C for 3 h	Nanospheres of 7–8 nm	0.01–3.0	Discharge: 1726 Charge: 1241 (50 mAg^−1^)	CE = 28.1% 1st cycle	1519 mAhg^−1^ at 50 mAg^−1^ after 10 cycles	[167]
SnO_2_	Tin acetate and sucrose in water	180 °C to evaporate then 450 °C for 3 h	Nanospheres of 12.5 nm	0.01–3.0	Discharge: ~850 Charge: ~750	CE = 92.7% 50th cycle	242 mAhg^−1^ at 50 mAg^−1^ after 50 cycles	[168]
Co-doped SnO_2_	Cobalt(II) gluconate, tin acetate and sucrose in water	180 °C to evaporate then 450 °C for 3 h	Nanospheres of 6.7–7.7–10.1 nm	0.01–3.0	Discharge: ~1200 Charge: ~1100	CE = 70.4% 1st cycle CE = 94.6–94.9% 50th cycle	493 mAhg^−1^ at 100 mAg^−1^ after 50 cycles 435.8 mAhg^−1^ at 50 mAg^−1^ after 50 cycles	[168]
Co-doped SnO_2_ with C coating	Cobalt(II) gluconate, tin acetate, sucrose and glucose in water	180 °C to evaporate then 450 °C for 3 h. Heat up again at 180 °C for 13 h	Nanospheres of ~10 nm embedded in carbon matrix	0.01–3.0	Discharge: ~1900 Charge: ~1700	CE = 74.2–74.4% 1st cycle CE = 96.0–97.2% 50th cycle	1000–1200 mAhg^−1^ at 50 mAg^−1^ after 50 cycles	[168]
SnO_2_	Na_2_SnO_3_·3H_2_O, urea in water	170 °C for 36 h then calcinated at 500 °C for 4 h	Shallow nanospheres of 500 nm and 38 nm thick	0.01–3.0	Discharge: 1203	CE = 61.6% 1st cycle	87 mAhg^−1^ at 100 mAg^−1^ after 300 cycles	[169]
Ni-doped SnO_2_	Na_2_SnO_3_·3H_2_O, urea, NiNO_3_ in ethanol/water	170 °C for 36 h then calcinated at 500 °C for 4 h	Shallow nanospheres of 500 nm and 20 nm thick	0.01–3.0	Discharge: 1463–1581	CE = 58.8–62.1% 1st cycle	542 mAhg^−1^ at 100 mAg^−1^ after 300 cycles	[169]
SnO_2_	SnO_2_ nanopowder	vapor deposition process at 1050 °C for 1 h 15 mbar	Nanowires of lengths 50 nm and 500 nm	0.005–2.5	Discharge: 612 Charge: 267 (1000 mAg^−1^)	CE = 43.6% first cycle	148 mAhg^−1^ at 1000 mAg^−1^ after 30 cycles	[170]
SnO_2_-Fe_2_O_3_	SnO_2_ nanopowder and FeCl_3_·6H_2_O	vapor deposition process at 1050 °C for 1 h 15 mbar	Nanowires with Fe_2_O_3_ nanoarrays	0.005–2.5	Discharge: 1167 Charge: 809 (1000 mAg^−1^)	CE = 69.4% first cycle	207 mAhg^−1^ at 1000 mAg^−1^ after 30 cycles	[170]
SnO_2_-graphene oxide-	SnCl_2_ and graphene oxide	Autoclave 220 °C 24 h	5–10 nm SnO_2_ nanoparticles	0.01–3.0	Discharge: 810 (100 mAg^−1^)	CR = 73.8% after 100 cycles	597 mAhg^−1^ at 100 mAg^−1^ after 100 cycles	[171]
SnO_2_-graphene oxide- Co_3_O_4_	SnCl_2_ and graphene oxide and Co(CH_3_COO)_2_	Autoclave 220 °C 24 h then 80 °C for 8 h	5–10 nm SnO_2_ and Co_3_O_4_ nanoparticles	0.01–3.0	Discharge: 1038 (1000 mAg^−1^)	CR = 100% after 100 cycles	1038 mAhg^−1^ at 100 mAg^−1^ after 100 cycles	[171]

## 6. Conclusions and Outlook

SnO_2_ is a versatile material whose bandgap can be modified by several strategies, including higher symmetry polymorph stabilization, doping and formation of defects in the structure through the synthesis process. Since the SnO_2_ compound is a potential candidate for applications in energy storage, studying SnO_2_ phase stabilization is, therefore, of interest. Amongst the available strategies, it has been shown that metal dopants can affect the morphology, decrease lattice parameters and simultaneously create oxygen-related defects. The use of metal dopants also tends to enhance the energy capacity, and when combined with a carbon matrix, e.g., graphene, graphite or carbon nanotubes, the reversibility of the reaction can be improved. In order to significantly improve the electrochemical properties of SnO_2_ nanostructures, the stabilization of higher symmetry polymorphs, i.e., high-pressure-induced phases, such as orthorhombic or cubic, is being intensively investigated. However, due to the difficulties encountered in stabilizing these phases, the electrochemical properties cannot be systematically probed. Nevertheless, the use of dopants such as nitrogen or metals to produce oxygen vacancies appears to be the most promising solution to stabilize the orthorhombic or cubic structures. However, the doping of SnO_2_ nanoparticles is still unable to stabilize single-phase SnO_2_, and several polymorphs tend to co-precipitate. However, in epitaxial thin films, the substrate-induced strain is capable of surpassing the activation free-energy barrier and leading to the stabilization of single-phase SnO_2_.

Today, there are several challenges related to introducing new nanomaterials for LiB electrode application. In particular, for the SnO_2_ compound, the main drawbacks, such as the huge initial capacity loss and extensive capacity fading after prolonged cycling, make their commercial breakthrough challenging compared to the well-established and omnipresent graphite. These drawbacks are mainly due to the volume expansion during charge–discharge processes that are still unaccounted for, despite consistent progress in the field. These stresses induced by the volume expansion have been alleviated to some extent by the addition of carbonaceous materials or by creating hollow structures that allow slightly higher flexibility for volume expansion. Therefore, hollow and hierarchical nanostructures are at the forefront of SnO_2_ research. Tailoring SnO_2_ nanostructures into desired morphologies is also a strategy to increase the active surface area, which in turn enhances the Li+ activity. In that regard, it has been shown that optimal electrochemical performances are obtained using ultra-small nanoparticles with a highly active specific surface. However, the scaling up of SnO_2_ synthesis processes in order to meet LiB demands needs to be cost effective and is one of the technical challenges to solve. Even if these aforementioned issues are surmountable, the technology transfer of nanomaterials to battery technologies involves many logistic issues. In fact, the weight of the active anode materials can attain 16% of the total battery weight in an electric vehicle, representing over 70 kg only for the anode material. Although SnO_2_ nanomaterials appear promising for increasing the general lifespan of a battery, their environmental impact is only succinctly addressed in the literature. The end-of-life recycling of materials has been shown to release free-standing ultrafine nanomaterials into the environment during the shredding process. Therefore, combining SnO_2_ nanoparticles with carbonaceous materials, including the presently employed graphite, could curb their release into the environment, in addition to improving the electrochemical properties of batteries. However, for a successful technology transfer, the sustainability of SnO_2_ is an important issue. In 2021, the production of Sn exceeded 370,000 tons. Being the 49th most-abundant element on Earth implies that Sn is quite scarce, and this would eventually lead to massive environmental and sustainability issues. To that end, growing nanostructures of SnO_2_ with large surface-to-volume ratios will be an advantage through the reduction in the volume of the material. This increases the sustainability of the SnO_2_ compound in LiB owing to the incorporation of lower quantities of Sn in the nanostructures.

Battery energy storage systems (BESS) are an important part of the net-zero energy transition. They have a wide range of power and storage capacities for both small-scale devices, including mobile phones, and large-scale devices in industrial utilities. LiBs have powered up to 90% of BESS globally. Present-day anodes employ cost-effective and light-weight carbon-based materials that tend to disintegrate after a finite number of charge–discharge cycles. In addition, nanomaterials have been proposed as anode materials to first overcome issues of volume expansion, which leads to the terminal degradation of the anode. Second, the high surface-to-volume ratio provided by nanostructured morphologies offers a higher number of active sites for Li storage that also facilitate reversible reactions. Therefore, integrating the SnO_2_ compound into the existing graphite-based anodes for LiB would offer a quick adaptation of this nanomaterial in battery applications. In fact, graphite-based anodes currently being employed already exhibit a strong network for SnO_2_–nanomaterial integration with a potential for a better battery cycle life and higher capacity, while making related processes more sustainable and cost effective.

## Figures and Tables

**Table 1:** Crystal structure of SnO_2_ polymorphs, volume of the unit cell (Å^3^), its density (in g.cm^−3^) and direct bandgaps (eV).

Structure	Symbol	Structure	Volume (Å^3^)		Density (g.cm^−3^)	Direct Bandgap (eV)	
			[87]	[88]	[87]	Cal. [51]	Exp.
Rutile	P42/mnm	Tetragonal	75.73	73.27	6.61	3.50	3.68 [42]
CaCl_2_	Pnnm	Orthorhombic	75.52	72.76	6.63	3.58	
α-PbO_2_	Pbcn	Orthorhombic	75.12	71.84	6.66	3.80	
Pyrite	Pa $\bar{3}$	Cubic	69.30	66.95	7.22	3.55	
ZrO_2_	Pbca	Orthorhombic	68.74	66.07	7.28	3.44	
Fluorite	Fm3m	Cubic	68.23	65.86	7.34	3.01	
Cotunnite	Pnam	Orthorhombic	69.25	63.76	7.23	2.84	4.1 [85]

## Data Availability

All figure were reproduced with permission or via creative commons attribution.
